# Case Report: Diffuse Polymicrogyria Associated With a Novel *ADGRG1* Variant

**DOI:** 10.3389/fped.2021.728077

**Published:** 2021-08-27

**Authors:** Fábio Carneiro, Júlia Duarte, Francisco Laranjeira, Sofia Barbosa-Gouveia, María-Luz Couce, Maria José Fonseca

**Affiliations:** ^1^Neurology Department, Hospital Garcia de Orta, Almada, Portugal; ^2^Neuroradiology Department, Hospital Garcia de Orta, Almada, Portugal; ^3^Biochemical Genetics Unit, Centro de Genética Médica Doutor Jacinto Magalhães, Porto, Portugal; ^4^Unit of Diagnosis and Treatment of Congenital Metabolic Diseases, Hospital Clínico Universitario de Santiago de Compostela, Instituto de Investigación Sanitaria de Santiago de Compostela, Centro de Investigación Biomédica en Red de Enfermedades Raras, European Reference Network for Rare Hereditary Metabolic Disorders, Santiago de Compostela, Spain; ^5^Child Development Centre Torrado da Silva, Hospital Garcia de Orta, Almada, Portugal

**Keywords:** *ADGRG1*, bilateral generalized polymicrogyria, *GPR56*, polymicrogyria, bilateral frontoparietal polymicrogyria, case report

## Abstract

Pathogenic variants of the *ADGRG1* gene are associated with bilateral frontoparietal polymicrogyria, defined radiologically by polymicrogyria with an anterior-posterior gradient, pontine and cerebellar hypoplasia and patchy white matter abnormalities. We report a novel homozygous *ADGRG1* variant with atypical features. The patient presented at 8 months of age with motor delay, esotropia, hypotonia with hyporeflexia and subsequently developed refractory epilepsy. At the last assessment, aged 12 years, head control, sitting and language were not acquired. Magnetic resonance imaging revealed diffuse polymicrogyria with relative sparing of the anterior temporal lobes, without an anterior-posterior gradient, diffuse hypomyelination and pontine and cerebellar hypoplasia. A panel targeting brain morphogenesis defects yielded an unreported homozygous *ADGRG1* nonsense variant (dbSNP rs746634404), present in the heterozygous state in both parents. We report a novel *ADGRG1* variant associated with diffuse polymicrogyria without an identifiable anterior-posterior gradient, diffuse hypomyelination and a severe motor and cognitive phenotype. Our case highlights the phenotypic diversity of *ADGRG1* pathogenic variants and the clinico-anatomical overlap between recognized polymicrogyria syndromes.

## Introduction

Polymicrogyria (PMG) is a cortical malformation characterized by supernumerary, small gyri with abnormal cortical lamination. PMG syndromes are markedly heterogeneous in etiology and phenotype, including infectious or vascular causes as well as genetically defined multiple congenital anomaly syndromes or inborn errors of metabolism. PMG can also occur in isolation (unilateral or bilateral PMG syndromes), frequently with a genetic etiology (1). Bilateral PMG syndromes are further categorized according to the topographical distribution of the abnormal gyral pattern into bilateral frontoparietal (BFPP), bilateral generalized (BGP), bilateral perisylvian, bilateral posterior or bilateral parasagittal PMG (1).

Autosomal recessive pathogenic variants of *GPR56* or *ADGRG1* (OMIM^*^604110; henceforth designated as *ADGRG1*), a member of the family of G protein–coupled receptors (GPCRs), were found to be specifically associated with BFPP (2, 3). BFPP is radiologically defined by the presence of PMG with an anterior to posterior gradient, bilateral patchy white matter signal changes and brainstem and cerebellar hypoplasia or dysplasia (3). The clinical phenotype consists of early onset hypotonia with subsequent motor and cognitive developmental delay, seizures and eye abnormalities (strabismus and/or nystagmus) (3). We herein report a novel *ADGRG1* variant causing a generalized PMG pattern and a severe clinical phenotype.

## Case Presentation

The proband is a 12-year-old male, the second-child of consanguineous parents (first degree cousins). The family history is relevant for learning disabilities and motor delay in a paternal aunt and learning disabilities in a paternal uncle. Notably, the patient's older brother by 9 years has no history of developmental delay or learning difficulties.

Gestation and birth were uneventful and in the post-natal period the patient only presented neonatal jaundice (maximal bilirubin dosing 12.4 mg/dL) treated with phototherapy.

**Figure 1:**
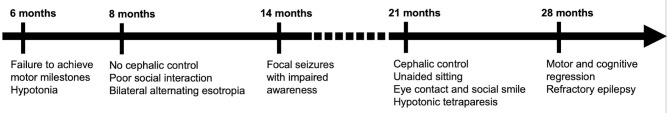
Timeline of symptoms and signs.

Global development was reportedly normal until 6 months of age, when hypotonia and failure to achieve motor milestones were first noticed and the patient was referred to our Child Neurology outpatient clinic (Figure 1). On first assessment, at 8 months of age, the general examination showed mild dysmorphic features (almond eyes, long philtrum and low-set ears) without other relevant findings. Neurological examination revealed bilateral alternating esotropia and predominantly upper limb and axial hypotonia with diminished reflexes. We found a significant delay in psychomotor development, cephalic control was not present and the patient was unable to sit. There was poor social interaction, only few vocalizations and no hand manipulation. The initial magnetic resonance imaging (MRI) at 9 months revealed diffuse polymicrogyria without an identifiable anterior-posterior gradient, diffuse hypomyelination, thin corpus callosum, supratentorial ventriculomegaly and pontine and cerebellar hypoplasia, affecting both cerebellar vermis and hemispheres. There were no abnormalities on the electroencephalogram (EEG). The patient underwent an evaluation for chromosomal abnormalities, fragile-X mutation, imprinting disorders, mitochondrial DNA and ATPase 6 mutations, comprehensive blood, urine and cerebrospinal fluid (CSF) metabolic screening and muscle biopsy with assessment of respiratory chain enzymatic activity, which were all normal. There was no evidence of systemic disease on echocardiogram, liver, renal and spleen ultrasounds.

At 14 months, the patient developed focal seizures with impaired consciousness (behavioral arrest and eye deviation) initially responsive to valproate. The EEG showed predominantly frontal spikes and spike-waves, within a normal background.

The patient progressed very slowly and was able to maintain cephalic control and an unaided sitting position at 21 months. He was able to maintain eye contact, smile and produce elementary sounds, but language was not present.

**Figure 2:**
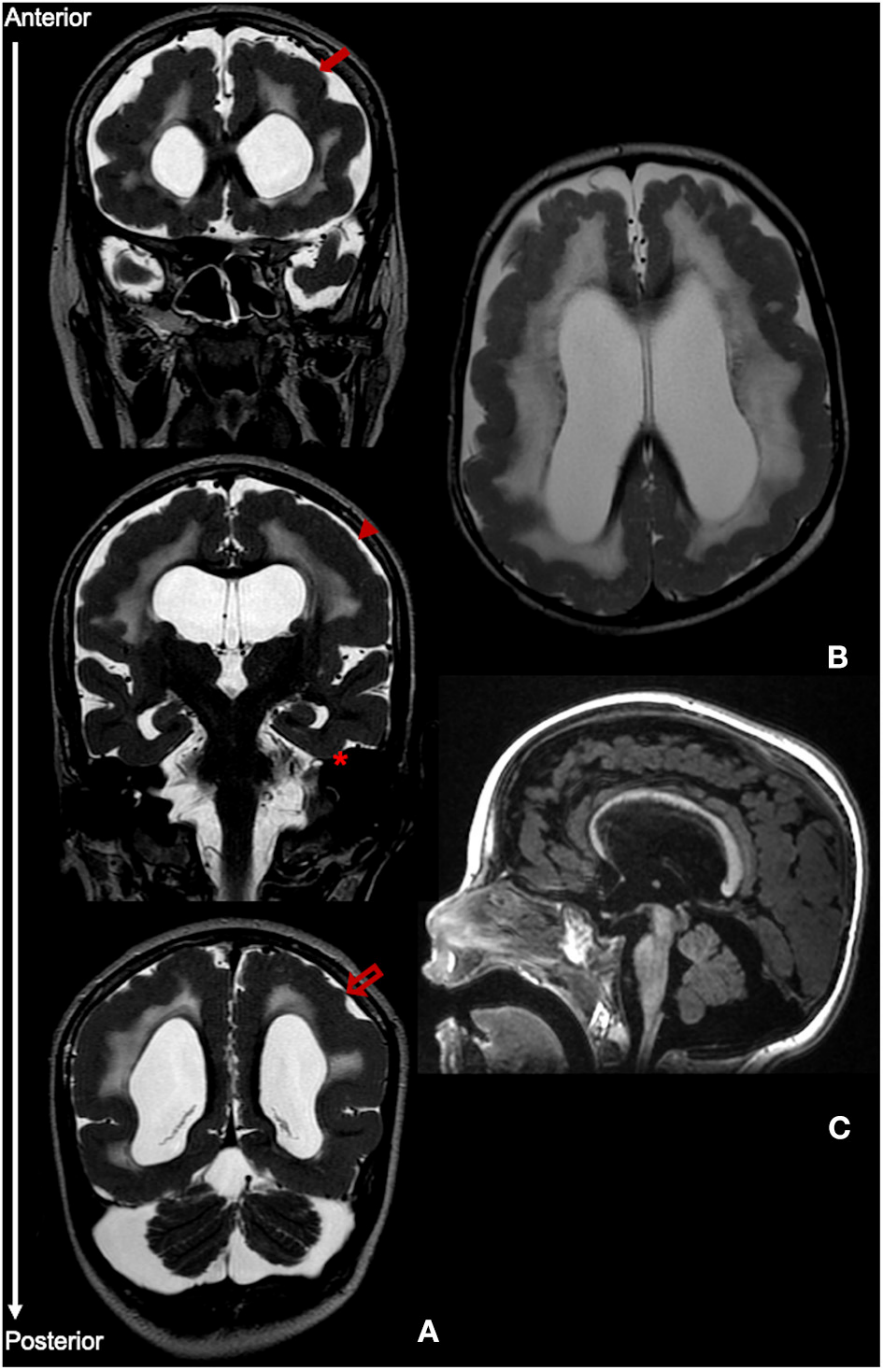
MRI findings at age 11 years. **(A)** Coronal T2-weighted images showing PMG without an anterior-posterior gradient of involvement, including frontal cortex (closed arrow), parietal cortex (arrowhead) and occipital cortex (open arrow). There is relative sparing of the temporal lobes (asterisk). **(B)** Axial T2 image displaying diffuse white matter hypomyelination. **(C)** Sagittal T1 image highlighting pontine and cerebellar hypoplasia.

At 2.5 years, however, there was significant motor and cognitive regression with loss of cephalic control and unsupported sitting ability and poorer interaction. Simultaneously, a gradual worsening of epilepsy was observed, with the appearance of multiple seizure types including epileptic spasms, atonic, myoclonic and tonic-clonic bilateral seizures. The EEG showed slow background activity with multifocal, anterior or centro-parietal predominant, spikes and spike-waves. The patient was subsequently treated with several anti-epileptics including vigabatrin, levetiracetam, topiramate, clonazepam, and perampanel, with partial control of seizures, but without significant motor and cognitive improvement. Serial neurological examinations consistently showed bilateral alternating esotropia, horizontal nystagmus and flaccid tetraparesis, without pyramidal or cerebellar signs. Visual and auditory evoked-potentials revealed prolonged conduction times bilaterally. Repeat MRI at 11 years of age (Figure 2) was globally consistent with the above-mentioned features.

**Figure 3:**
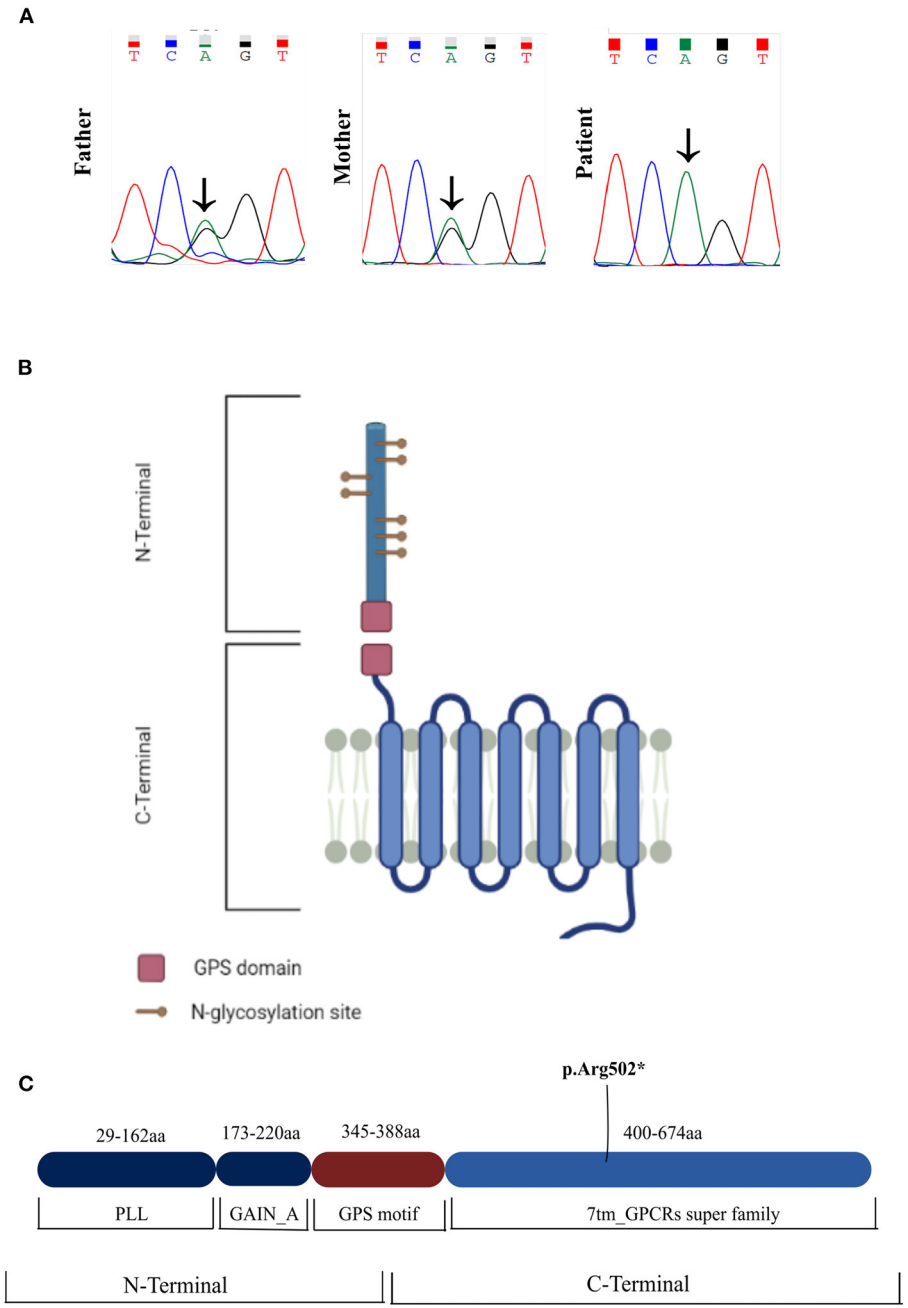
**(A)** Sanger sequencing chromatograms of the patient and parents. The patient is a homozygous carrier of the nonsense variant c.1504C>T. The variant was present in the heterozygous state in both parents. **(B,C)** Schematic of the ADGRG1 protein, showcasing the novel variant in the 7TM domain of the protein (p.Arg502*). 7TM, 7 transmembrane domain; GAIN, GPCR-Autoproteolysis-INducing domain; GPS, GPCR proteolysis site motif; PLL, Pentraxin/Laminin/neurexin/sex-hormone-binding-globulin-Like domain.

Genetic analysis with a next-generation sequencing panel targeting brain morphogenesis defects yielded a homozygous *ADGRG1* nonsense variant (rs746634404), caused by a nucleotide change [(NM_001145771.2: c.1504C>T) (NP_ 005673.3: p.Arg502Ter)] in exon 12. This variant is not present homozygously in GnomAD. Segregation analysis through Sanger sequencing confirmed the presence of the variant in the homozygous state in the patient and revealed that both parents were heterozygous carriers (Figure 3A). The variant is located in the seven-transmembrane (7TM) domain of the *ADGRG1* protein, which binds to a heterotrimeric G-protein complex and initiates downstream signaling (Figures 3B,C). The reported allele is rare in the population [GnomAD frequency: *f* = 0.0000119; European (Non-Finnish): *f* = 0.00000881]. *In silico* analysis was performed using two different algorithms to predict the pathogenicity of the variant. DANN (4) (Deleterious Annotation of genetic variants using Neural Networks) is a pathogenicity scoring methodology based on neural networks, with values ranging from 0 to 1 (where 1 represents variants predicted to be most damaging). MutationTaster (5) employs a Bayes classifier to predict the disease potential of an alteration, based on evolutionary conservation, splice-site, mRNA, protein and regulatory features. The reported variant was predicted as “damaging” by DANN (DANN score: 0.9983) and as “disease causing” by MutationTaster. Finally, the level of evidence of pathogenicity was classified as “very strong” according to American College of Medical Genetics criteria (6), as the nonsense variant was identified in a gene for which loss-of-function is a known mechanism of disease.

## Discussion

We report a novel *ADGRG1* truncating variant, presenting with atypically diffuse PMG and hypomyelination and a severe clinical phenotype.

At the time of publication, *ADGRG1* pathogenic variants have been described in a total of 77 patients from 47 pedigrees and 34 different corresponding variants (2, 3, 7–20). In all reported cases, *ADGRG1* variants were associated with a typical anatomical BFPP distribution, i.e., either a restricted frontoparietal PMG or an anterior-posterior gradient of cortical malformation. However, in our case a gradient of involvement was not identified. Complete or near-complete involvement of the entire cerebral cortex without any region of maximal involvement or any gradient of severity is the hallmark of BGP (21, 22). Relative sparing of the interhemispheric or temporal gyri has been described occasionally and is present in our patient. Considering other associated imaging features, patchy white matter signal changes or white matter volume reduction are most commonly associated with BFPP while more diffuse white matter involvement is present in more than 50% of BGP patients (22). The most distinguishing feature, however, is the presence of brainstem and cerebellar involvement in BFPP, also identified in our patient, and typically absent in BGP. Therefore, we believe that the anatomical distribution of cortical and non-cortical dysgenesis in our case mostly conforms to a severe and diffuse form of the BFPP syndrome.

Clinically, BFPP presents as a pseudomyopathic pattern, with hypotonia developing in the first year of life and occasionally being identified at birth (13). This initial clinical phenotype and imaging pattern is frequently suggestive of congenital muscular dystrophies of the “cobblestone complex” (13). The initial course of disease in our patient mimicked this pattern, but similarly to previously reported cases the muscle biopsy was normal and thus channeled the investigation away from this group of diseases. Following the pseudomyopathic presentation, the clinical course of BFPP is also considerably homogeneous in the cases reported in literature. Table 1 describes the frequency of clinical features associated with BFPP described in the reported cases.

**Table 1:** Clinical features of reported BFPP cases (2, 3, 8–20).

**Clinical feature**	***n* (%)**
**Motor impairment**
Able to walk	40 (81.6)
Age at walking in years, median (IQR)	3.5 (3.0)
Unable to walk	9 (18.4)
Missing	25
**Cognitive impairment**
Severe	46 (79.3)
Moderate	11 (18.9)
Mild	1 (1.7)
Missing	16
**Cerebellar signs**
Present	50 (92.6)
Absent	4 (7.4)
Missing	18
**Pyramidal signs**
Present	44 (75.9)
Absent	14 (24.1)
Missing	16
**Oculomotor findings**
Present	59 (92.1)
Strabismus	25 (59.5)
Nystagmus	6 (14.3)
Strabismus + nystagmus	10 (23.8)
Other^a^	1 (2.4)
Missing	17
Absent	5 (7.9)
Missing	10
**Head circumference**
Normal	57 (85.1)
Microcephaly	6 (9.0)
Macrocephaly	4 (5.9)
Missing	7
**Seizures**
Present	60 (88.2)
Age at onset in years, median (IQR)	3.0 (3.0)
Refractory	36 (60.0)
Missing	13
Absent	8 (11.8)
Missing	6

a *Macular pigmentary changes; IQR, interquartile range*.

Cognitive and motor delay are universal features, although their severity is variable between cases. While the majority of patients (79.3%) had severe cognitive impairment with language restricted to only a few words, motor impairment was reportedly milder. Indeed, most patients (81.6%) were able to walk, either with or without support, although gait is frequently described as “unsteady” or ataxic. Gait acquisition was delayed in almost all cases (13, 20). On the other hand, there was a total of nine cases without gait acquisition, originating from six different variants in seven pedigrees, of which five variants were either frameshift or nonsense (14, 20). In our case, also a nonsense variant, motor impairment was abnormally severe, since the patient was unable to walk, sit or achieve cephalic control. To our knowledge, there are only two reports sharing this severe phenotype. One is curiously of a Portuguese patient described by Santos-Silva et al. though the two cases do not share the same variant (14). The other report, by Cauley et al. describes a family with severe motor and cognitive delay, where the typical imaging phenotype of BFPP was present in association with Joubert-like features (7). The patients were found to have an additional pathogenic variant in *KIAA0556* by exome sequencing, with both variants interacting to create a more severe and complex phenotype. Pyramidal signs and ataxia are also common (75.9 and 92.6%), although pyramidal signs were absent in our patient and ataxia was impossible to ascertain due to the severity of motor impairment. Other common clinical manifestations in BFPP and present in this case are epilepsy (88.2% of patients described in the literature, being drug-refractory in 60% of those) and oculomotor dysfunction (92.1% of reported cases, most commonly strabismus). In short, the clinical picture present in our case is in accordance to BFPP, although the severity of motor impairment is atypical.

Could this severe phenotype be more in line with BGP? In the study by Leventer et al. (22), BGP patients were more likely to present with global developmental delay, intellectual disability, spastic quadriplegia, seizure onset at younger age, and hearing and cortical visual impairment, when compared to other PMG syndromes. Interestingly, the reported severity of cognitive and motor delay was also variable, with mild impairment in some cases, although severe phenotypes similar to our patient were reported (21). Thus, there seems to be considerable clinical overlap between BFPP and BGP in both the type and severity of clinical manifestations.

Several hypotheses can be proposed to explain the atypical clinical features in our case. First, they may result from the unique molecular characteristics of the gene product associated with the pathogenic variant. Physiologically, the ADGRG1 protein is a GPCR that binds extracellular matrix proteins (most notably collagen type III) and undergoes agonist-induced conformational changes. This, in turn, promotes release of the α subunit of the heterotrimeric G-protein complex from the 7TM domain of ADGRG1 and initiates downstream signaling via interaction with various cytosolic effector proteins. Even though other pathogenic variants of the surrounding 7TM region (such as the L487P, E496K, and R565W variants) do not present this particular phenotype (8, 12, 18), it is possible that subtle variations in the mutated protein impair different downstream signaling cascades, thereby resulting in varied phenotypic expressions. In our particular case, the severity of the clinical phenotype can also be interpreted in the context of an epileptic encephalopathy since there was motor and cognitive regression occurring simultaneously with epilepsy decompensation. Another hypothesis is the co-existence of additional pathogenic variants of genes involved in brain morphogenesis, as highlighted by the recent report from Cauley et al.

## Conclusions

We report a novel *ADGRG1* truncating variant associated with generalized PMG without an anterior-posterior gradient, diffuse hypomyelination and a severe motor phenotype. Clinical and radiological features of BFPP were also present, highlighting the clinical-anatomical overlap between recognized PMG syndromes and the phenotypic diversity of *ADGRG1* pathogenic variants.

## Data Availability Statement

The original contributions presented in the study are included in the article/supplementary materials, further inquiries can be directed to the corresponding author/s.

## Ethics Statement

The studies involving human participants were reviewed and approved by Ethics Committee of Clinical Research of Galicia (2015/410). Written informed consent to participate in this study was provided by the participants' legal guardian/next of kin.

## Author Contributions

FC and MF contributed to conception and design of the study. SB-G and M-LC performed the genetic experiments and data analysis. FC wrote the first draft of the manuscript. FC and SB-G designed the figures. All authors contributed to clinical care of the patient, manuscript revision, read, and approved the submitted version.

## Conflict of Interest

The authors declare that the research was conducted in the absence of any commercial or financial relationships that could be construed as a potential conflict of interest.

## Publisher's Note

All claims expressed in this article are solely those of the authors and do not necessarily represent those of their affiliated organizations, or those of the publisher, the editors and the reviewers. Any product that may be evaluated in this article, or claim that may be made by its manufacturer, is not guaranteed or endorsed by the publisher.
